# Iron overload is associated with increased susceptibility to pulmonary *Pythium insidiosum* infection in mice

**DOI:** 10.1371/journal.pntd.0014246

**Published:** 2026-04-24

**Authors:** Donglin Zhu, Ling Yan, Jian Sun, Shaoban Chen, Yun Xi, Kouxing Zhang, Mingliang Li

**Affiliations:** 1 Department of Clinical Laboratory, The Third Affiliated Hospital of Sun Yat-Sen University, Guangzhou, China; 2 Department of General ICU, The Third Affiliated Hospital of Sun Yat-Sen University, Guangzhou, China; 3 National Risk Assessment Laboratory for Antimicrobial Resistance of Animal Original Bacteria, South China Agricultural University, Guangzhou, China; Albert Einstein College of Medicine, UNITED STATES OF AMERICA

## Abstract

**Background:**

*Pythium insidiosum* is a fungus-like oomycete capable of causing disease with high mortality rates. Although pulmonary *Pythium insidiosum* infections are rare, they are associated with an extremely high fatality rate. The mechanisms underlying *Pythium insidiosum* infection remain unclear and may be associated with immune dysregulation or iron overload. Existing animal models do not adequately replicate the pathophysiological process of pulmonary invasive infection by *Pythium insidiosum* in humans. Therefore, this study aims to establish a murine model of pulmonary *Pythium insidiosum* infection and to investigate the role of the Th1/Th2 immune balance in the progression of pulmonary infection.

**Methods:**

BALB/c mice were divided into the following groups: control group, iron dextran (ID) group, cyclophosphamide (CTX) group, lipopolysaccharide (LPS) group, and groups affected by various factor combinations (e.g., LPS + CTX group, ID + CTX group, ID + LPS group). Mice were inoculated intratracheally with *Pythium insidiosum* hyphae after pretreatment. Body weight, clinical symptoms, inflammatory cell counts in venous blood and bronchoalveolar lavage fluid (BALF), pulmonary histopathological damage, pathogen burden, as well as serum levels of ferritin (FER), Th1 cytokines (IL-2, IL-12p70, IFN-γ, TNF-α) and Th2 cytokines (IL-4, IL-5, IL-13, IL-10) were assessed in each group.

**Results:**

Among all groups, LPS + CTX and ID + CTX groups exhibited severe infection symptoms, significant weight loss, and the highest clinical symptom scores (all *P* < 0.001). The ID + CTX group showed more pronounced increases in hematoxylin eosin (HE)-stained lung injury scores and Grocott’s Methenamine Silver (GMS)-stained hyphal burden scores (all *P* < 0.001). Compared with the control group, the ID + CTX group demonstrated decreased venous blood leukocyte (WBC), neutrophil (NEUT), and lymphocyte (LYMPH) counts, elevated FER levels, and significantly reduced LYMPH counts in BALF (all *P* < 0.01). Cytokine analysis revealed that Th1 cytokine (IFN-γ, TNF-α) levels were significantly suppressed in the ID + CTX group after preconditioning (both *P* < 0.001). Following infection, Th1 responses remained relatively suppressed, while Th2 responses showed an upward trend that did not reach statistical significance (all *P* > 0.05), more consistent with a relative Th2 shift resulting from Th1 suppression.

**Conclusion:**

This study established a murine model of pulmonary *Pythium insidiosum* infection for the first time through intratracheal hyphae inoculation following ID + CTX preconditioning. The findings suggest that iron overload is associated with Th1 immune response suppression and relative Th2 shift, which may be related to increased susceptibility and pathological damage in pulmonary *Pythium insidiosum* infection. However, further functional experiments are needed to validate causal relationships.

## Author summary

*Pythium insidiosum* is a dangerous pathogen that causes pythiosis, a life-threatening disease affecting humans and animals in tropical and subtropical regions. Although pulmonary infections are extremely rare, they are almost always fatal. The reasons why some individuals develop severe infections remain poorly understood. Based on clinical observations of patients with thalassemia, a condition associated with both iron overload and immune abnormalities, we hypothesized that iron overload might increase susceptibility to *Pythium insidiosum* infection by disrupting the delicate balance between two key immune responses: Th1 (which fights intracellular pathogens) and Th2 (which targets parasites). In this study, we established the first mouse model of pulmonary *Pythium insidiosum* infection and found that mice with iron overload combined with immunosuppression developed the most severe infections, with significant lung damage and high fungal burden. These mice showed suppressed Th1 immune responses and a relative shift toward Th2 responses. Our findings suggest that iron overload may increase susceptibility to pulmonary pythiosis through immune modulation, providing important insights for managing infections in patients with iron overload conditions such as thalassemia. Further research is needed to develop targeted immunomodulatory therapies.

## 1. Introduction

*Pythium insidiosum* is an aquatic fungus-like oomycete and the sole causative agent of pythiosis. This disease is endemic in tropical and subtropical regions, capable of infecting both humans and animals, leading to cutaneous, ocular, vascular, or even systemic invasive infections with extremely high mortality rates [1–3]. Its ecological distribution has shown a trend of expansion, which may be associated with global warming. The lungs are the most common target organ for invasive fungal infections, and once involved, the prognosis is exceedingly poor [4–7]. Notably, the reported mortality rate in the four naturally occurring cases of animal pulmonary pythiosis reached 100%, yet the mechanisms of infection remain unclear to date [8–11].

Previously, two rare cases of human deep pythiosis were reported by our group, suggesting that immunocompromised status and iron overload may be risk factors for pythiosis infection [12,13]. Iron overload typically suppresses cellular immunity (Th1 response) while potentially promoting humoral immunity (Th2 response), thereby shifting the immune balance towards a Th2 bias [14–16]. In fungal infections, the Th1 response generally plays a protective role, whereas the Th2 response is often associated with disease progression [17,18]. Based on this, we hypothesize that the Th1/Th2 imbalance induced by iron overload may trigger and exacerbate the onset and progression of *Pythium insidiosum* infection.

To date, the role and mechanism of the Th1/Th2 immune balance in pulmonary *Pythium insidiosum* infection have never been elucidated. BALB/c mice, known for their extensive use and reproducibility in immunological studies and various pathogen infection models, were selected as the model animal for this study. The aim was to establish a murine model of pulmonary *Pythium insidiosum* infection, to investigate whether iron overload can trigger infection by inducing a Th1/Th2 imbalance, and to explore the key role of this immune imbalance in driving pulmonary immunopathological injury and disease progression.

## 2. Materials and methods

### 2.1. Ethics statement

SPF female BALB/c mice (6–8 weeks old) were purchased from the Guangdong Provincial Medical Laboratory Animal Center. All experiments were performed in compliance with the relevant laws and regulations on the use and management of laboratory animals in China. The study was reviewed and approved by the Experimental Animal Ethics Committee of South China Agricultural University (Approval No. 2020C407).

### 2.2. Experimental materials

The *Pythium insidiosum* strain used in this study was provided by the Microbiology Laboratory of the Third Affiliated Hospital of Sun Yat-sen University. The strain was cultured on Luria-Bertani (LB) agar plate at 37°C for 96 hours. Mycelia were collected, washed with sterile distilled water, and homogenized to prepare a uniform mycelial suspension as the inoculum for the animal model. Optical density was measured spectrophotometrically, and the inoculum was adjusted to 70%-75% transmittance at 600 nm. To test inoculum viability, 100 μL of the suspension was re-cultured on LB agar plate at 37°C for 48 hours before inoculation to observe colony formation; simultaneously, suspension uniformity was assessed microscopically to ensure a highly purified, viable, and homogeneous suspension without mycelial clumps.

### 2.3. Establishing Pulmonary *Pythium Insidiosum* infection model

According to different pretreatment protocols, the experiment was divided into 7 groups with 10 mice in each group.

(1) LPS group: LPS (5 mg/kg in 100 μL) via intratracheal intubation on day 4 and 1 prior to hyphal inoculation.
(2) CTX group: CTX (100 μL) via intraperitoneal injection with doses of 150 mg/kg on day 4 and 100 mg/kg on day 1 prior to hyphal inoculation, respectively.
(3) ID group: ID (375 mg/kg in 100 μL) via intraperitoneal injection every 2 days, starting from day 7 before hyphal inoculation.
(4) LPS + CTX group: Received both LPS and CTX preconditioning.
(5) ID + CTX group: Received both ID and CTX preconditioning.
(6) ID + LPS group: Received both ID and LPS preconditioning.
(7) Control group: Received an equal volume of normal saline.

After preconditioning, all mice were inoculated intratracheally with *Pythium insidiosum* hyphal suspension (100 μL/mouse).

### 2.4. Assessment of clinical status

At different stages of the study, including baseline (study initiation), post-preconditioning (24 hours before hyphal inoculation), and infection endpoint (48 hours after hyphal inoculation), observations and recordings were performed daily before feeding. Body weight of mice in each group was measured, and clinical symptoms (activity behavior, hair ruffling, stool consistency) were quantified using a scoring system with the following detailed criteria:

(1) Activity behavior: 1 = normal activity, active exploration, normal gait, responsive; 2 = reduced activity, still able to move, slightly sluggish reaction; 3 = significantly reduced activity, unwilling to move, curled up in corner, able to move upon stimulation, unsteady gait; 4 = nearly immobile, moribund state, or exhibiting trembling, spinning, and other abnormal behaviors.
(2) Hair ruffling: 1 = smooth, close-fitting, shiny fur; 2 = mildly erect fur, loss of luster, but still basically close-fitting; 3 = obvious erect fur, fluffy, poor luster, visible skin folds; 4 = severely erect and dry fur, noticeably wrinkled and loose skin.
(3) Stool consistency: 1 = normally formed, moderately firm and dry, granular; 2 = mildly soft stool, slightly altered shape but still formable; 3 = moderate diarrhea, unformed stool, paste-like, possible contaminating perianal area; 4 = severe diarrhea, watery stool, or accompanied by mucus streaks, severe contamination of perianal area and tail.

The three scores were summed to obtain the total clinical symptom score, used for comprehensive assessment of the overall clinical status of mice.

### 2.5. Evaluation of inflammatory cell counts

Venous blood samples were collected from all groups at baseline, post-preconditioning, and infection endpoint. WBC, NEUT, and LYMPH counts were analyzed using hematology analyzer. BALF samples were collected at the experimental endpoint, and NEUT and LYMPH counts were determined using cell counter.

### 2.6. Histopathological and etiological assessment

At infection endpoint, lung tissues from all groups were subjected to HE stain for lung injury scoring (based on the method established by the American Thoracic Society Animal Acute Lung Injury Model Evaluation Panel [19]). Simultaneously, a semi-quantitative scoring system was used to assess hyphal burden in GMS-stained lung tissue sections: 0 = no hyphae, 1 = few scattered hyphae, 2 = moderate hyphal infiltration, 3 = extensive hyphal aggregation.

### 2.7. Assessment of immune response levels

Venous blood samples were collected from all groups at baseline, post-preconditioning, and infection endpoint. Serum FER levels and immune-related cytokines, including Th1 cytokines (IL-2, IL-12p70, IFN-γ, TNF-α) and Th2 cytokines (IL-4, IL-5, IL-10, IL-13), were measured by ELISA.

### 2.8. Statistical analysis

Statistical analysis was performed using SPSS 25.0 software. Quantitative data are presented as mean ± standard deviation (Mean ± SD). Comparisons among multiple groups were performed using one-way analysis of variance (ANOVA). If differences were statistically significant (*P* < 0.05), Bonferroni correction was further applied for post-hoc pairwise comparisons to determine differences.

## 3. Results

### 3.1. Clinical Manifestations (Fig 1)

At baseline, there were no statistically significant differences in body weight (*F* = 1.894, *P* = 0.117) or clinical symptom scores (*F* < 0.001, *P* = 1.000) among mice in the control, LPS, CTX, ID, LPS + CTX, ID + CTX, and ID + LPS groups.

**Fig 1:**
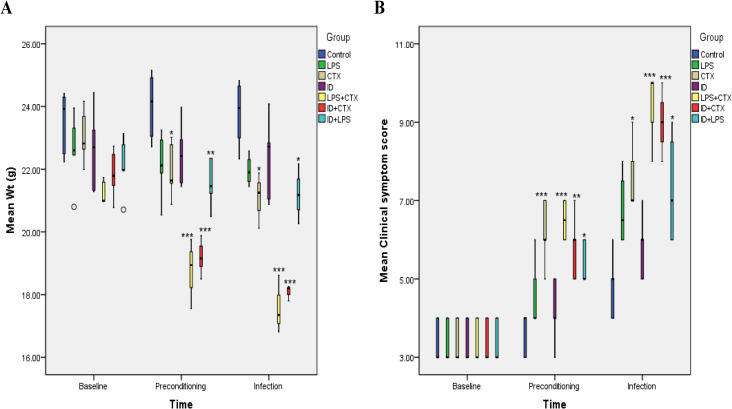
Effects of different pretreatments on body weight (A) and clinical symptom scores (B) in mice infected with *Pythium insidiosum.* Results showed that at the end of preconditioning and infection endpoint, CTX, LPS + CTX, ID + CTX, and ID + LPS groups had significantly decreased body weight and significantly increased clinical symptom scores compared with the control group, with the most pronounced changes in LPS + CTX and ID + CTX groups (*P < 0.05, **P < 0.01, ***P < 0.001).

At the end of preconditioning, significant differences were observed among groups in body weight (*F* = 18.585, P < 0.001) and clinical symptom scores (*F* = 9.979, *P* < 0.001). Specific results were as follows:

(1) Compared with the control group, CTX, LPS + CTX, ID + CTX, and ID + LPS groups showed decreased body weight, with statistically significant differences (*P* = 0.037, < 0.001, < 0.001, = 0.006, respectively); LPS and ID groups showed no statistically significant differences in body weight (*P* = 0.077, 0.298, respectively).
(2) Compared with the control group, CTX, LPS + CTX, ID + CTX, and ID + LPS groups showed progressively sluggish reactions, disheveled and dull hair, loose stool consistency, and increased clinical symptom scores, with statistically significant differences (*P* < 0.001, < 0.001, = 0.002, = 0.015, respectively); LPS and ID groups showed no statistically significant differences in scores (*P* = 0.908, 1.000, respectively).

At the infection endpoint, significant differences were observed among groups in body weight (*F* = 23.216, *P* < 0.001) and clinical symptom scores (*F* = 10.230, *P* < 0.001). Specific results were as follows:

(1) Compared with the control group, CTX, LPS + CTX, ID + CTX, and ID + LPS groups showed decreased body weight, with statistically significant differences (*P* = 0.015, < 0.001, < 0.001, = 0.01, respectively); LPS and ID groups showed no statistically significant differences in body weight (*P* = 0.178, 0.462, respectively).
(2) Compared with the control group, CTX, LPS + CTX, ID + CTX, and ID + LPS groups showed progressively irritable reactions, erect hair, diarrhea in some cases, and increased clinical symptom scores, with statistically significant differences (*P* = 0.022, < 0.001, < 0.001, = 0.041, respectively); LPS and ID groups showed no statistically significant differences in scores (*P* = 0.219, 1.000, respectively).

### 3.2. Blood Inflammatory Cells (Fig 2)

At baseline, no statistically significant differences were observed among the groups in WBC (*F* = 0.568, *P* = 0.752), NEUT (*F* = 0.949, *P* = 0.477), or LYMPH (*F* = 0.348, *P* = 0.905) counts.

**Fig 2:**
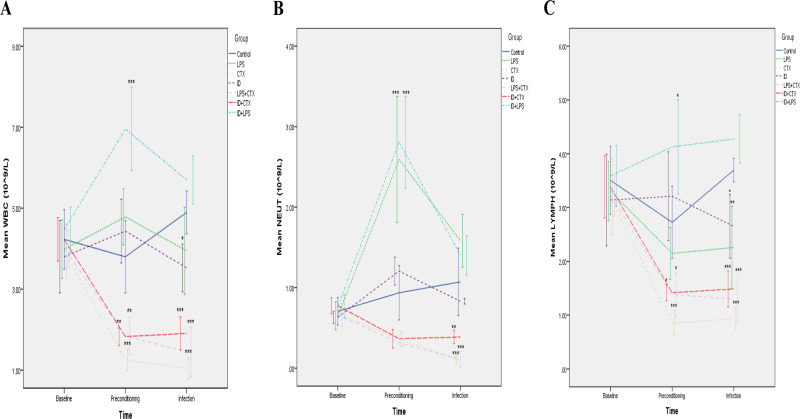
Effects of different pretreatments on venous blood inflammatory cell counts in mice infected with *Pythium insidiosum.* Overall trends showed that compared with the control group, the ID + CTX group had decreased WBC **(A)**, NEUT **(B)**, and LYMPH (C) counts at the end of preconditioning and infection endpoint, suggesting an immunosuppressive state (*P < 0.05, **P < 0.01, ***P < 0.001).

Following the preconditioning period, significant differences emerged in WBC (*F* = 42.769, *P* < 0.001), NEUT (*F* = 33.576, *P* < 0.001), and LYMPH (*F* = 19.131, *P* < 0.001) counts. Compared with the control group, the results of the inflammatory cell counts in the remaining groups of mice are as follows:

(1) WBC counts in the CTX, LPS + CTX, and ID + CTX groups exhibited significantly lower (*P* < 0.001, = 0.004, = 0.002, respectively), whereas the ID + LPS group showed a significant increase (*P* < 0.001), while no statistically significant differences in the LPS and ID groups (*P* = 0.304, 0.771, respectively).
(2) NEUT counts were significantly elevated in the LPS and ID + LPS groups (both *P* < 0.001), while no statistically significant differences in the CTX, ID, LPS + CTX, and ID + CTX groups (*P* = 0.299, 0.937, 0.287, 0.314, respectively).
(3) LYMPH counts in the CTX, LPS + CTX, and ID + CTX groups were significantly reduced (*P* < 0.001, = 0.03, = 0.023, respectively), but elevated in the ID + LPS group (*P* = 0.012), while no statistically significant differences in the LPS and ID groups (*P* = 0.711, 0.843, respectively).

At the infection endpoint, significant intergroup differences persisted for WBC (*F* = 29.351, *P* < 0.001), NEUT (*F* = 21.758, *P* < 0.001), and LYMPH (*F* = 26.273, *P* < 0.001) counts. Compared with the control group, the results of the inflammatory cell counts in the remaining groups of mice are as follows:

(1) WBC counts were significantly lower in the CTX, ID, LPS + CTX, and ID + CTX groups (*P* < 0.001, = 0.034, < 0.001, < 0.001, respectively), while no statistically significant differences in the LPS and ID + LPS groups (*P* = 0.318, 0.509, respectively).
(2) NEUT counts were significantly decreased in the CTX, LPS + CTX, and ID + CTX groups (*P* < 0.001, < 0.001, = 0.007, respectively), while no statistically significant differences in the LPS, ID and ID + LPS groups (*P* = 0.066, 0.707, 0.438, respectively).
(3) LYMPH counts were significantly reduced in the LPS, CTX, ID, LPS + CTX, and ID + CTX groups (*P* = 0.003, < 0.001, = 0.032, < 0.001, < 0.001, respectively), while no statistically significant difference in the ID + LPS group (*P* = 0.56).

### 3.3. BALF inflammatory cells

**Fig 3:**
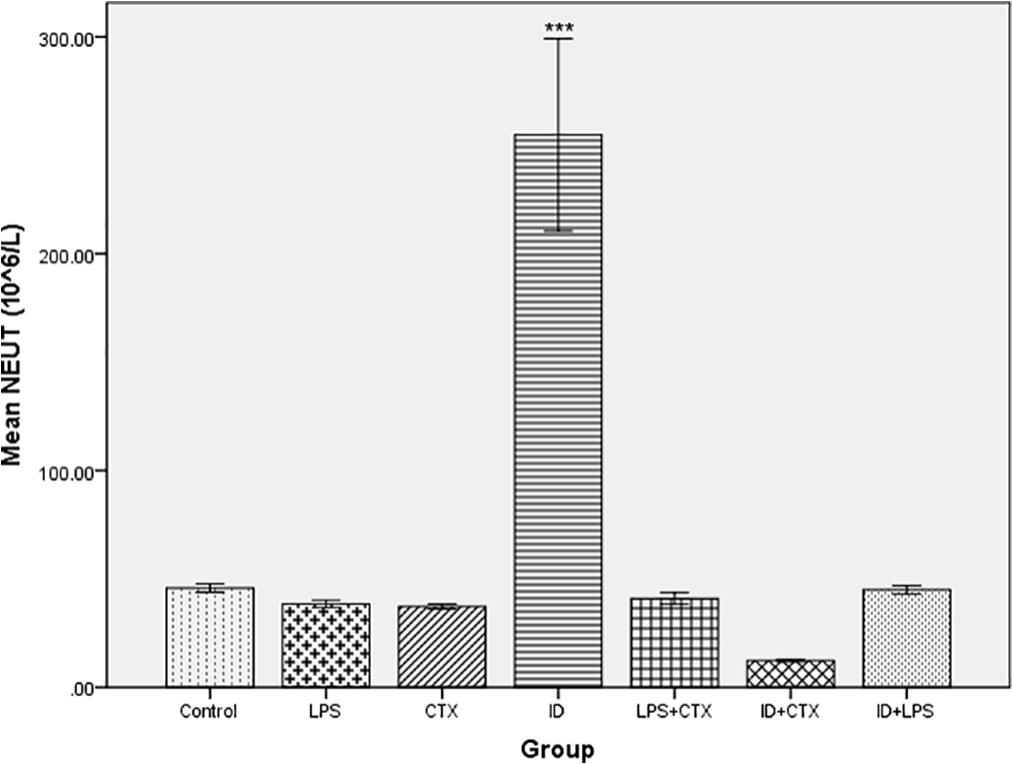
Effects of different pretreatments on BALF NEUT counts in mice infected with *Pythium insidiosum.* Compared with the control group, only the ID group showed significantly increased NEUT counts, while other groups showed no statistical differences (*P < 0.05, **P < 0.01, ***P < 0.001).

Significant differences in BALF NEUT counts (×10⁶/L) were observed among the groups (*F* = 77.01, *P* < 0.001). Cell counts, in ascending order, were: ID + CTX group (12.33 ± 0.58), CTX group (37.33 ± 1.15), LPS group (38.50 ± 1.73), LPS + CTX group (41.00 ± 2.65), ID + LPS group (45.00 ± 2.00), control group (45.80 ± 1.92), and ID group (254.80 ± 44.40). Compared with the control group, the ID group exhibited significantly elevated NEUT counts (*P* < 0.001), while no significant differences were observed in the LPS, CTX, LPS + CTX, ID + CTX, and ID + LPS groups (*P* = 0.998, 0.997, 1.00, 0.32, 1.00, respectively) (Fig 3).

**Fig 4:**
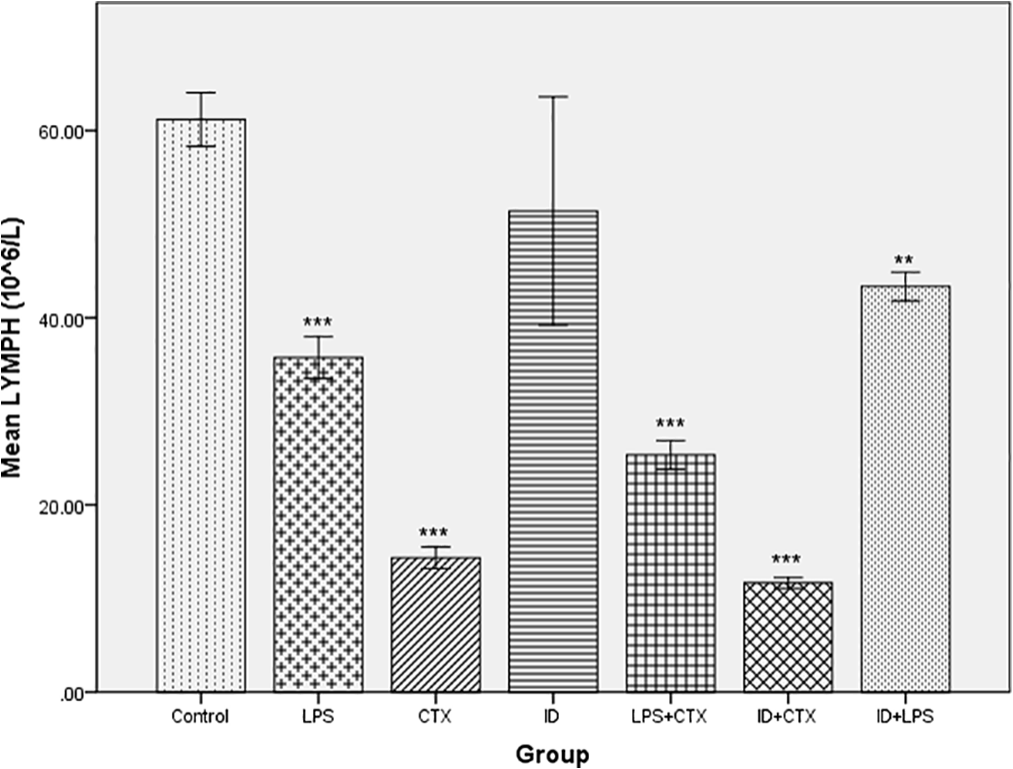
Effects of different pretreatments on BALF LYMPH counts in mice infected with *Pythium insidiosum.* Compared with the control group, LPS, CTX, LPS + CTX, ID + CTX, and ID + LPS groups showed significantly decreased LYMPH counts, while the ID group showed no statistical difference (*P < 0.05, **P < 0.01, ***P < 0.001).

Significant differences in BALF LYMPH counts (×10⁶/L) were observed among the groups (*F* = 38.23, *P* < 0.001). The counts in ascending order were: ID + CTX (11.67 ± 0.58), CTX (14.33 ± 1.15), LPS + CTX (25.33 ± 1.53), LPS (35.75 ± 2.22), ID + LPS (43.33 ± 1.53), ID (51.40 ± 12.22), and control (61.20 ± 2.86). Compared with the control group, significantly lower LYMPH counts were observed in the LPS, CTX, LPS + CTX, ID + CTX, and ID + LPS groups (*P* < 0.001, < 0.001, < 0.001, < 0.001, = 0.008, respectively), while no significant difference was found in the ID group (*P* = 0.17) (Fig 4).

### 3.4. Pulmonary histopathological damage

**Fig 5:**
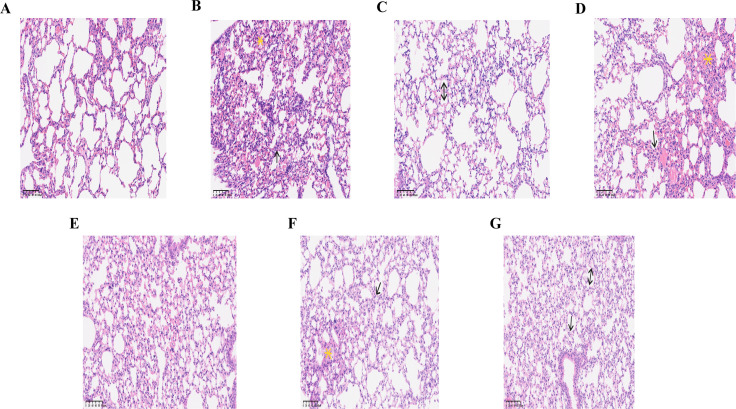
Effects of different pretreatments on lung tissue pathology in mice infected with *Pythium insidiosum* (HE staining, 300 × , scale bar = 100 μm). Control group (A) showed clear and intact alveolar structure, slightly septal thickening, and no infiltration in alveolar spaces. LPS group (B) showed alveolar structural disruption, septal thickening (single arrow), and inflammatory cells and erythrocytes infiltration in alveolar spaces (yellow asterisk). CTX group (C) showed alveolar structural disorder, septal destruction, and local hyaline membrane formation (double arrows) in some areas. ID group (D) showed obvious alveolar septal thickening (single arrow), and some alveolar spaces filled with inflammatory cells, erythrocytes, and protein debris (yellow asterisk). LPS + CTX group (E) showed severely disrupted alveolar structure but relatively mild alveolar septal thickening, with slightly inflammatory exudation. ID + CTX group (F) showed local alveolar structural disruption, septal thickening (single arrow), significant inflammatory cells infiltration, and a few erythrocytes and protein debris in alveolar spaces (yellow asterisk). ID + LPS group (G) showed alveolar structural disruption with mild alveolar septal thickening (single arrow), scattered inflammatory cells and erythrocytes in alveolar spaces, and hyaline membrane formation (double arrows) in some areas.

HE stains revealed distinct pulmonary histopathological changes across groups. Control group maintained essentially normal alveolar architecture with slight septal thickening and absence of inflammatory cells or erythrocytes. LPS group showed structural disruption with dense inflammatory infiltration with abundant neutrophils, lymphocytes, and erythrocytes in alveolar spaces. CTX group demonstrated marked structural damage, scattered inflammatory cells, and local hyaline membrane formation. ID group displayed significant septal thickening with inflammatory infiltration, and some alveolar spaces contained inflammatory cells, erythrocytes, and protein debris. LPS + CTX group had substantial structural destruction but milder septal thickening, with few inflammatory cells and protein debris. ID + CTX group displayed partial structural damage, septal thickening, mixed inflammatory cell infiltration, along with erythrocytes and protein debris. ID + LPS group showed structural damage with mild septal thickening, scattered inflammatory cells and erythrocytes, and hyaline membrane formation (Fig 5).

**Fig 6:**
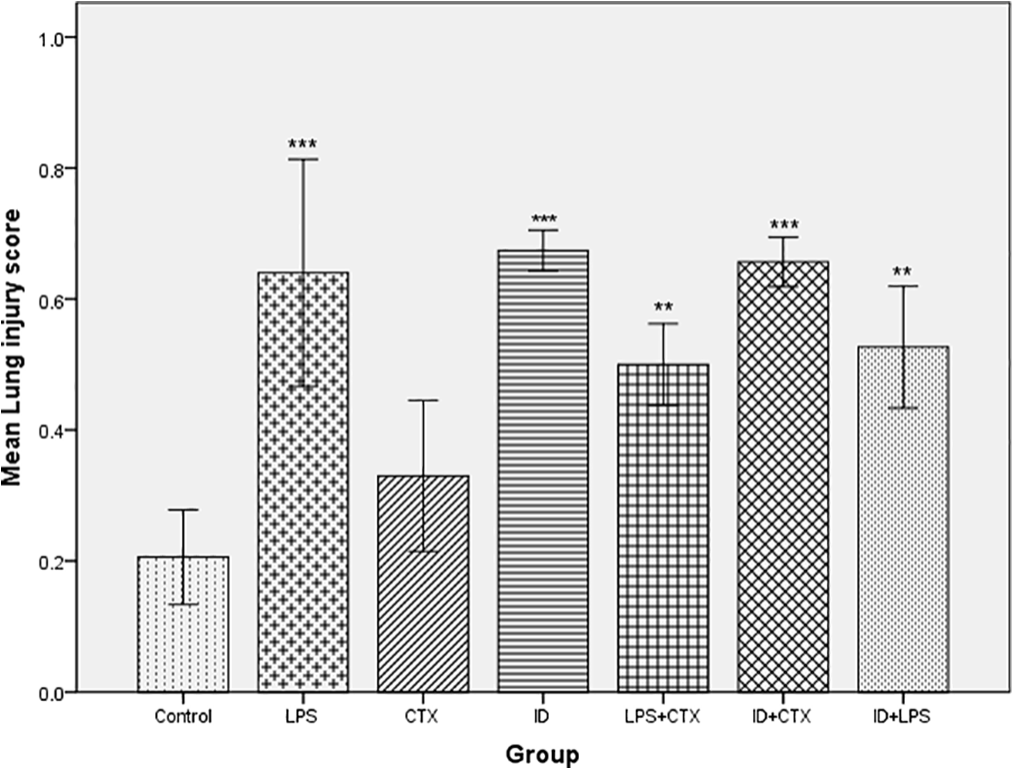
Effects of different pretreatments on lung injury scores in mice infected with *Pythium insidiosum.* Compared with the control group, LPS, ID, LPS + CTX, ID + CTX, and ID + LPS groups showed significantly increased lung injury scores, with relatively higher injury scores and more severe damage in LPS, ID + CTX, and ID groups (*P < 0.05, **P < 0.01, ***P < 0.001).

Significant differences in lung injury scores were observed among the groups (*F* = 15.396, *P* < 0.001). Lung injury scores across groups in increasing order were: control (0.21 ± 0.072), CTX (0.33 ± 0.115), LPS + CTX (0.50 ± 0.062), ID + LPS (0.53 ± 0.093), LPS (0.64 ± 0.173), ID + CTX (0.66 ± 0.038), and ID (0.67 ± 0.031). Compared with the control group, significantly higher scores were observed in the LPS, ID, LPS + CTX, ID + CTX, and ID + LPS groups (*P* < 0.001, < 0.001, = 0.006, < 0.001, = 0.003, respectively), while the CTX group showed no significant difference (*P* = 0.565) (Fig 6).

### 3.5. Assessment of pathogen burden in lung tissue

**Fig 7:**
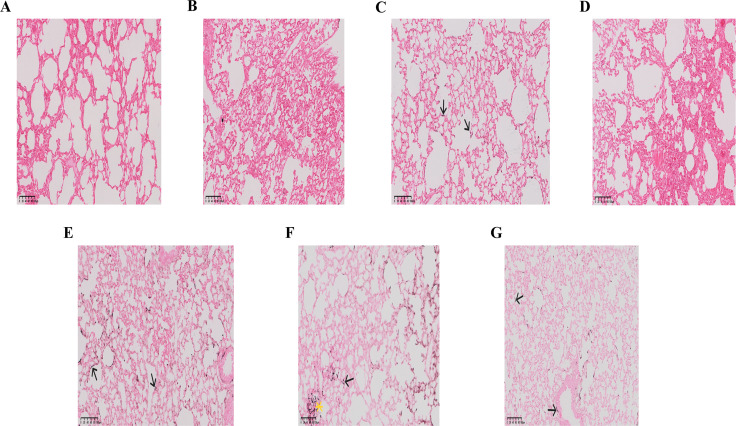
Effects of different pretreatments on lung tissue hyphal burden in mice infected with with *Pythium insidiosum* (GMS staining, 300 × , scale bar = 100 μm). Control group **(A)**, LPS group **(B)**, and ID group (D) showed no black hyphal structures in lung tissue sections. CTX group **(C)**, ID + LPS group **(G)**, and LPS + CTX group (E) showed a little slender, septate black hyphae scattered in alveolar spaces and interstitium (arrow). ID + CTX group (F) showed extensive distribution of numerous black hyphae in lung tissues (arrow) with local hyphal clumping or aggregation (asterisk).

GMS stains were employed to evaluate hyphal burden in mouse lung tissues. No hyphae were detected in the control, LPS, or ID groups. The CTX group showed sparse, thin, septate black hyphae with uniform width distributed in alveolar spaces, interstitium, and around bronchi. The LPS + CTX and ID + LPS groups exhibited similar but scanty black hyphal fragments or spores around alveolar septa and perivascular areas. In contrast, the ID + CTX group demonstrated numerous widely distributed hyphae with localized aggregation (Fig 7).

**Fig 8:**
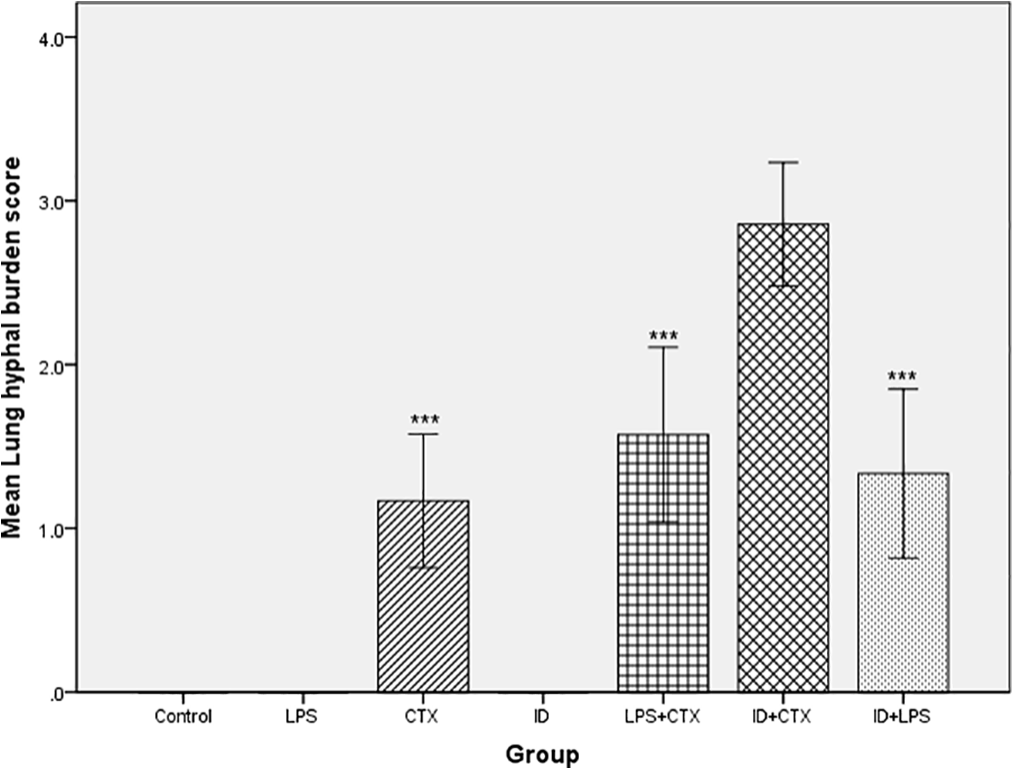
Effects of different pretreatments on lung hyphal burden scores in mice infected with *Pythium insidiosum.* Control, LPS, and ID groups had no detectable hyphae. Compared with the ID + CTX group with the heaviest hyphal burden, CTX, LPS + CTX, and ID + LPS groups showed significantly decreased hyphal burden scores (*P < 0.05, **P < 0.01, ***P < 0.001).

Hyphal burden scores for groups with detectable hyphae, sorted from lowest to highest, were: CTX group (1.167 ± 0.408), ID + LPS group (1.333 ± 0.516), LPS + CTX group (1.571 ± 0.535), ID + CTX group (2.857 ± 0.378). Comparison of lung hyphal burden scores among groups with detectable hyphae revealed statistically significant differences (*F* = 46.852, *P* < 0.001). Compared with the ID + CTX group, CTX, ID + LPS, and LPS + CTX groups showed decreased lung hyphal burden scores, with statistically significant differences (all *P* < 0.001) (Fig 8).

### 3.6. Serum FER Levels (Fig 9)

At baseline, no significant differences in FER levels were detected among the groups (*F* = 0.768, *P* = 0.607).

**Fig 9:**
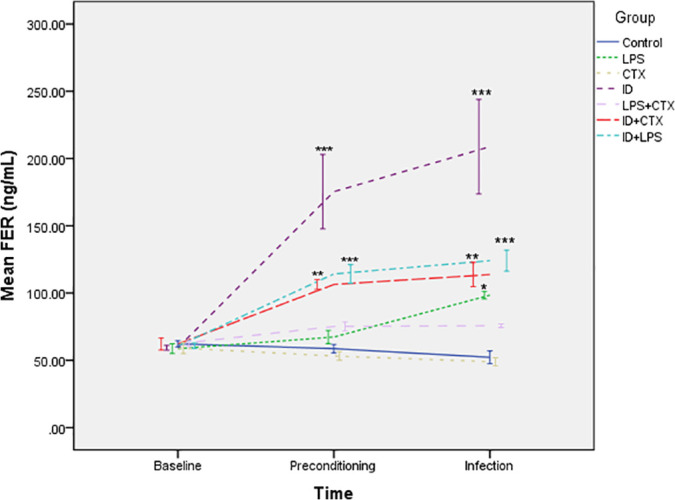
Effects of different pretreatments on serum ferritin levels in mice infected with *Pythium insidiosum.* Overall trends showed that at the end of preconditioning and infection endpoint, all groups involving ID pretreatment (ID, ID + CTX, ID + LPS groups) had significantly higher serum ferritin levels compared with the control group, successfully simulating an iron overload state (*P < 0.05, **P < 0.01, ***P < 0.001).

Following preconditioning, significant intergroup differences emerged (*F* = 44.337, *P* < 0.001). The ID, ID + CTX, and ID + LPS groups showed significantly elevated FER levels compared with controls (*P* < 0.001, = 0.002, < 0.001, respectively), while no significant differences were observed in the LPS, CTX, and LPS + CTX groups (*P* = 0.958, 0.996, 0.567, respectively).

At the infection endpoint, significant differences persisted (*F* = 44.629, *P* < 0.001). The LPS, ID, ID + CTX, and ID + LPS groups maintained significantly higher FER levels versus controls (*P* = 0.018, < 0.001, = 0.002, < 0.001, respectively), while the CTX and LPS + CTX groups showed no significant differences(*P* = 1.00, 0.445, respectively).

### 3.7. Serum Th1 Cytokine Levels (Fig 10)

At baseline, no significant differences were detected in IL-2 (*F* = 0.067, *P* = 0.998), IL-12p70 (*F* = 0.263, *P* = 0.946), IFN-γ (*F* = 0.284, *P* = 0.935), or TNF-α (*F* = 0.090, *P* = 0.996) levels among groups.

**Fig 10:**
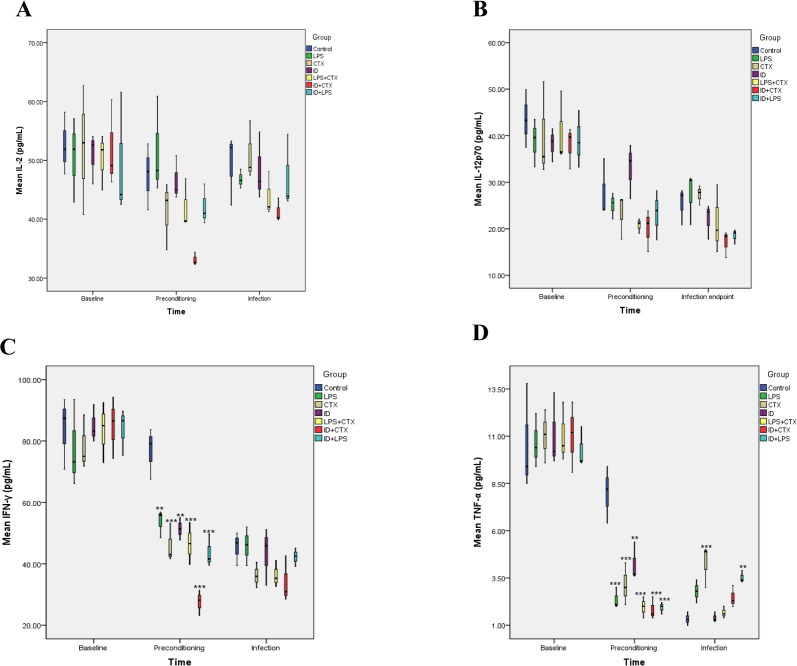
Effects of different pretreatments on serum Th1 cytokine levels in mice infected with *Pythium insidiosum.* IL-2 (A) and IL-12p70 (B) showed no statistically significant differences among groups. At the end of preconditioning, compared with the control group, all treatment groups showed significantly suppressed IFN-γ (C) and TNF-α (D) levels, particularly pronounced in ID-related groups. At the infection endpoint, TNF-α showed significant recovery in CTX and ID + LPS groups, but remained suppressed in the ID + CTX group (*P < 0.05, **P < 0.01, ***P < 0.001).

Following preconditioning, significant intergroup differences emerged for IL-2 (*F* = 4.029, *P* = 0.015), IFN-γ (*F* = 19.468, P < 0.001), and TNF-α (*F* = 18.623, *P* < 0.001) levels. In addition, IL-12p70 levels remained unchanged across all groups (*F* = 2.648, *P* = 0.062). Compared with the control group, the results of the Th1 cytokines levels in the remaining groups of mice are as follows:

(1) IL-2 levels showed no significant differences in any experimental group (all *P* > 0.05).
(2) IL-12p70 levels showed no significant differences in any experimental group (all *P* > 0.05).
(3) IFN-γ levels were significantly reduced in the LPS, CTX, ID, LPS + CTX, ID + CTX, and ID + LPS groups (*P* = 0.005, < 0.001, = 0.002, < 0.001, < 0.001, < 0.001, respectively).
(4) TNF-α levels were significantly decreased in the LPS, CTX, ID, LPS + CTX, ID + CTX, and ID + LPS groups (*P* < 0.001, < 0.001, = 0.003, < 0.001, < 0.001, < 0.001, respectively).

At the infection endpoint, significant intergroup differences emerged for TNF-α levels (*F* = 11.448, *P* < 0.001). In addition, IL-2 (*F* = 1.494, *P* = 0.250), IL-12p70 (*F* = 2.725, *P* = 0.057), and IFN-γ (*F* = 1.943, *P* = 0.143) levels demonstrated no significant intergroup variations. Compared with the control group, the results of the Th1 cytokines levels in the remaining groups of mice are as follows:

(1) IL-2 levels showed no significant differences in any experimental group (all *P* > 0.05).
(2) IL-12p70 levels showed no significant differences in any experimental group (all *P* > 0.05).
(3) IFN-γ levels showed no significant differences in any experimental group (all *P* > 0.05).
(4) TNF-α levels were significantly elevated in the CTX (*P* < 0.001) and ID + LPS (*P* = 0.008) groups, while the LPS (*P* = 0.163), ID (*P* = 1.000), LPS + CTX (*P* = 1.000), and ID + CTX (*P* = 0.649) groups showed no significant differences.

### 3.8. Serum Th2 Cytokine Levels (Fig 11)

At baseline, no significant differences were detected in IL-4 (*F* = 0.688, *P* = 0.663), IL-5 (*F* = 0.623, *P* = 0.709), IL-10 (*F* = 0.666, *P* = 0.679), or IL-13 (*F* = 0.617, *P* = 0.714) levels among groups.

**Fig 11:**
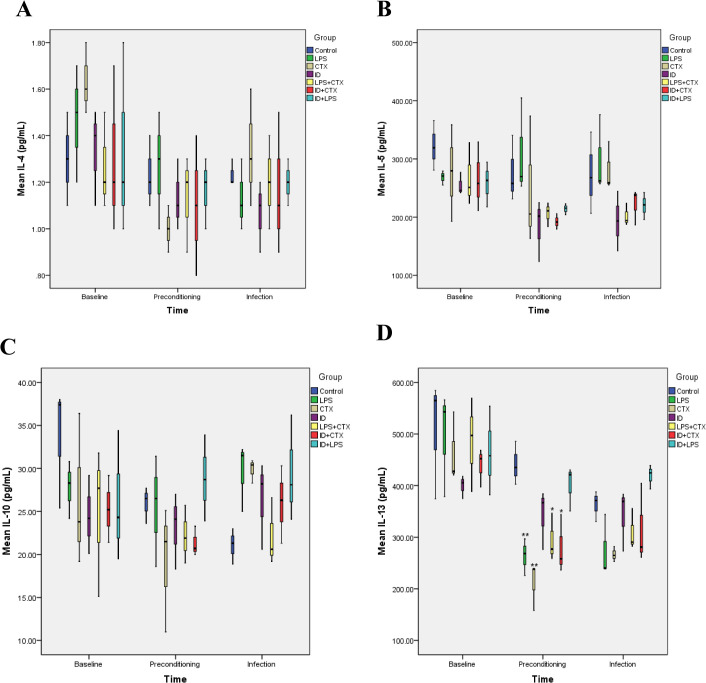
Effects of different pretreatments on serum Th2 cytokine levels in mice infected with *Pythium insidiosum.* At the end of preconditioning, IL-4 **(A)**, IL-5 **(B)**, and IL-10 (C) showed no significant differences among groups; LPS, CTX, LPS + CTX, and ID + CTX groups showed significantly decreased IL-13 (D) levels compared with the control group. At the infection endpoint, all Th2 factors showed slight upward trends among groups, but the differences were not statistically significant (*P < 0.05, **P < 0.01, ***P < 0.001).

Following preconditioning, only IL-13 showed significant differences (*F* = 8.608, *P* < 0.001), with no significant differences detected in IL-4 (*F* = 0.586, *P* = 0.736), IL-5 (*F* = 1.761, *P* = 0.179), or IL-10 (*F* = 1.389, *P* = 0.286) levels among groups. Compared with the control group, the results of the Th2 cytokines levels in the remaining groups of mice are as follows:

(1) IL-4 levels showed no significant differences in any experimental group (all *P* > 0.05).
(2) IL-5 levels showed no significant differences in any experimental group (all *P* > 0.05).
(3) IL-10 levels showed no significant differences in any experimental group (all *P* > 0.05).
(4) IL-13 levels were significantly reduced in the LPS, CTX, LPS + CTX, and ID + CTX groups (*P* = 0.009, 0.001, 0.042, 0.020, respectively), while the ID (*P* = 0.493) and ID + LPS (*P* = 1.000) groups showed no significant differences

At the infection endpoint, significant intergroup differences emerged for IL-13 levels (*F* = 3.581, *P* = 0.023). In addition, IL-4 (*F* = 0.567, *P* = 0.750), IL-5 (*F* = 2.379, *P* = 0.085), and IL-10 (*F* = 2.250, *P* = 0.099) levels demonstrated no significant intergroup variations. Compared with the control group, the results of the Th2 cytokines levels in the remaining groups of mice are as follows:

(1) IL-4 levels showed no significant differences in any experimental group (all *P* > 0.05).
(2) IL-5 levels showed no significant differences in any experimental group (all *P* > 0.05).
(3) IL-10 levels showed no significant differences in any experimental group (all *P* > 0.05).
(4) IL-13 levels showed no significant differences in any experimental group (all *P* > 0.05).

## 4. Discussion

*Pythium insidiosum*, a fungus-like oomycete, which is a rare but serious pathogen in tropical and subtropical regions, causes pythiosis, a disease that infects both humans and animals and is characterized by high mortality [1–3]. In clinical practice, the lungs are a primary concern in invasive fungal infections; invasion of pulmonary tissues by fungal pathogens generally leads to poor patient prognosis, severe complications, and life-threatening conditions [4,5]. Over recent decades, global warming has altered the ecological niches of numerous saprophytic and environmental fungi, increasing their interaction with human activities. This shift has contributed to the emergence of novel fungus-like pathogens worldwide, resulting in invasive pulmonary infections that pose significant threats to human health [6,7]. Although only four natural cases of pulmonary pythiosis in animals have been reported (all of which were fatal), and no human cases have been documented to date, the fungus-like characteristics of *Pythium insidiosum* highlight its substantial potential for pulmonary invasion, underscoring the need for vigilance against this pathogen [8–11].

Previous clinical cases reported by our group described two rare cases of human deep pythiosis: one involving soft tissue infection and another spinal infection. Both patients had concurrent thalassemia with iron overload and immune abnormalities [12,13]. This aligns with reports from some researchers that thalassemia can drive iron overload through chronic hemolysis, exacerbating oxidative stress and inflammation, mutually inhibiting immune function, and disrupting the Th1/Th2 balance, thereby increasing susceptibility to opportunistic infections [14–16]. The Th1/Th2 balance of helper T cells represents a cornerstone of adaptive immunity: Th1 responses primarily mediate cellular immunity to eliminate intracellular pathogens, while Th2 responses promote antibody production against parasitic infections, though their overactivation may lead to fibrosis and immunosuppression. In fungal infections, Th1 responses typically confer protection, whereas Th2 responses are associated with disease progression [17,18]. Based on this, we hypothesize that iron overload-induced Th1/Th2 imbalance may be a significant risk factor for *Pythium insidiosum* infection.

Current understanding of the infection mechanisms and effective treatments for *Pythium insidiosum* remain unclear, with limited available animal studies, predominantly focusing on skin and soft tissue infections [1–3]. However, the most common site of invasive fungal infection in clinical practice is not skin and soft tissue, but rather the lungs [4,5]. Existing pythiosis models cannot adequately replicate the pathophysiological process of pulmonary invasive infection by *Pythium insidiosum* in humans, resulting in significant limitations in clinical translation of experimental findings [3]. To address this gap, this study establishes a murine model of pulmonary *Pythium insidiosum* infection and comprehensively explores the impact of iron overload on Th1/Th2 immune balance and its role in the initiation and progression of pulmonary infection. Our results demonstrate that iron overload preconditioning significantly enhances host susceptibility to *Pythium insidiosum* and exacerbates pulmonary immunopathological damage by disrupting Th1/Th2 immune balance.

To further explore the mechanisms of *Pythium insidiosum* pulmonary infection from the perspective of Th1/Th2 immune dysregulation, we simulated clinically relevant iron overload conditions (such as in thalassemia patients) through ID preconditioning. Notably, ID preconditioning alone caused no significant weight loss, but combined ID + CTX preconditioning produced the most severe clinical manifestations, weight reduction, and pulmonary damage. Particularly noteworthy is that the ID + CTX group exhibited the most severe pulmonary hyphal burden, suggesting a synergistic effect between iron overload and immunosuppression that significantly enhances the colonization and invasive capability of *Pythium insidiosum* in the lungs. These findings are highly consistent with the poor prognosis observed in clinical patients with combined iron overload and immunosuppression [20–22]. It must be acknowledged that although the study used semi-quantitative scoring to assess lung tissue hyphal burden, it still lacks more precise quantitative methods (such as qPCR), which is an area for improvement in future research.

Through dynamic monitoring of inflammatory cells counts during infection progression, we observed that the ID + CTX group maintained persistently low LYMPH counts in both peripheral blood and BALF throughout the infection course, concurrently demonstrating the most severe pulmonary tissue damage and hyphal burden. This indicates a strong correlation between reduced LYMPH counts and failure in infection control, supporting the crucial role of Th1 cell-mediated immune responses in combating *Pythium insidiosum* infection, while their suppression directly impairs pathogen clearance capacity [23]. Furthermore, analysis of inflammatory cells in BALF revealed significantly elevated NEUT counts in the ID group, suggesting dysregulated local inflammatory responses potentially related to Th2 immune skewing-induced immunomodulatory disruption, which may further compromise pulmonary barriers and facilitate hyphal invasion.

To further elucidate the specific mechanisms by which Th1/Th2 balance-mediated adaptive immunity regulates pulmonary *Pythium insidiosum* infection, this study systematically analyzed dynamic changes in serum Th1 and Th2 cytokines levels throughout the infection course. Following preconditioning, ID-related groups (ID, ID + CTX, ID + LPS) exhibited significantly elevated serum FER levels accompanied by suppressed Th1 cytokines (including IL-2, IL-12p70, IFN-γ, and TNF-α), indicating that iron overload induces systemic suppression of Th1 immunity. Although partial recovery of certain Th1 cytokines were observed post-infection in some groups (notably IL-2, IL-12p70, and TNF-α in CTX group; IL-12p70 and TNF-α in LPS group), the overall Th1 response remained relatively suppressed in ID-related groups. Conversely, Th2 cytokines (IL-4, IL-5, IL-10, IL-13), initially suppressed during preconditioning, demonstrated elevated though not statistically significant levels post-infection. These trends were particularly pronounced in ID + CTX and CTX groups, and when correlated with their severe infection phenotypes, suggesting that dysregulated Th2 responses may contribute to infection progression under immunosuppressive conditions. Our data showed that after Pythium insidiosum infection, Th1 cytokines (such as IFN-γ) were suppressed, but Th2 cytokines (such as IL-4, IL-5, IL-10) did not show statistically significant increases. This relative Th2 shift, formed against a background of Th1 suppression rather than absolute Th2 polarization, may be associated with disease progression.

In summary, this study establishes the first murine model of pulmonary *Pythium insidiosum* infection and demonstrates that iron overload disrupts immune balance by suppressing Th1 responses and promoting a relative Th2 skewing, thereby creating a favorable immune microenvironment for *Pythium insidiosum* infection. This significantly increases susceptibility to pulmonary infection, disrupts local inflammatory regulation, and leads to immunopathological damage. These mechanisms not only deepen our understanding of the pathogenesis of *Pythium insidiosum* infection but also provide potential immunomodulatory targets for anti-infective therapy in clinical patients with iron overload (such as thalassemia or transfusion-related iron overload).

However, it should be pointed out that there are some shortcomings in this study. Firstly, the BALB/c mouse strain used in this study has an inherent Th2 bias, which may have amplified the observed “relative Th2 shift” phenomenon to some extent, limiting the extrapolation of conclusions to hosts with other genetic backgrounds. Future research should validate our findings in Th1-biased C57BL/6 mice or other strains. Secondly, this study primarily demonstrated associations among iron overload, immune changes and infection susceptibility, rather than direct causal relationships. The lack of functional validation experiments such as iron chelator intervention or immune cell adoptive transfer is one of the limitations of our study. To further elucidate the underlying mechanisms, subsequent studies should investigate the therapeutic potential of iron chelators, Th1 cytokines agonists and immune checkpoint modulators in such infections, aiming to restore immune balance for improved infection control and clinical outcomes.
